# Assessment of Awareness, Perceptions, and Opinions towards Artificial Intelligence among Healthcare Students in Riyadh, Saudi Arabia

**DOI:** 10.3390/medicina59050828

**Published:** 2023-04-24

**Authors:** Wajid Syed, Mahmood Basil A. Al-Rawi

**Affiliations:** 1 Department of Clinical Pharmacy, College of Pharmacy, King Saud University, Riyadh 11451, Saudi Arabia; 2 Department of Optometry, College of Applied Medical Sciences, King Saud University, Riyadh 11451, Saudi Arabia

**Keywords:** artificial intelligence, robots, awareness, perceptions, opinions, pharmacy, students, Saudi Arabia

## Abstract

*Background and Objective*: The role of the pharmacist in healthcare society is unique, since they are providers of health information and medication counseling to patients. Hence, this study aimed to evaluate Awareness, Perceptions, and Opinions towards Artificial intelligence (AI) among pharmacy undergraduate students at King Saud University (KSU), Riyadh, Saudi Arabia. *Materials and Methods:* A cross-sectional, questionnaire-based study was conducted between December 2022 and January 2023 using online questionnaires. The data collection was carried out using convenience sampling methods among senior pharmacy students at the College of Pharmacy, King Saud University. Statistical Package for the Social Sciences version 26 was used to analyze the data (SPSS). *Results*: A total of one hundred and fifty-seven pharmacy students completed the questionnaires. Of these, most of them (*n* = 118; 75.2%) were males. About 42%, (*n* = 65) were in their fourth year of study. Most of the students (*n* = 116; 73.9%) knew about AI. In addition, 69.4% (*n* = 109) of the students thought that AI is a tool that helps healthcare professionals (HCP). However, more than half 57.3% (*n* = 90) of the students were aware that AI would assist healthcare professionals in becoming better with the widespread use of AI. Furthermore, 75.1% of the students agreed that AI reduces errors in medical practice. The mean positive perception score was 29.8 (SD = 9.63; range-0–38). The mean score was significantly associated with age (*p* = 0.030), year of study *(p* = 0.040), and nationality (*p* = 0.013). The gender of the participants was found to have no significant association with the mean positive perception score (*p* = 0.916). *Conclusions*: Overall, pharmacy students showed good awareness of AI in Saudi Arabia. Moreover, the majority of the students had positive perceptions about the concepts, benefits, and implementation of AI. Moreover, most students indicated that there is a need for more education and training in the field of AI. Consequently, early exposure to content related to AI in the curriculum of pharmacy is an important step to help in the wide use of these technologies in the graduates’ future careers.

## 1. Introduction

The global healthcare system has received a great deal of attention as a result of the utilization of cutting-edge technologies to deliver the greatest caliber of healthcare. This includes artificial intelligence (AI) [1,2,3]. The theory and development of computer systems that can do activities that ordinarily require human intellect, such as speech recognition, visual perception, decision-making, and language translation, are referred to as artificial intelligence [4] This indicates that AI functions similarly to humans but not exactly as humans do; this function is still in development [5,6]. Data science and information technology are undergoing a revolution owing to artificial intelligence (AI), which advances automated tasking technologies [5,6].

Nevertheless, AI is classified into two groups: weak AI and strong AI, where weak AI systems are those that are tailored for certain tasks [7]. They may detect things that are comparable to what they know and label them accordingly. This provides a human-like experience, yet it is only a simulation [7]. The AI may not understand your commands, but it will respond to them using algorithms. Apple’s Siri, which uses the Internet as a powerful database, is an excellent example of a weak AI. Strong AI, on the other hand, is a machine that can mislead a person into thinking it is likewise human. Strong AI is a machine that can experience awareness [7]. They are thought to possess human cognitive capacities [7]. When a powerful AI is confronted with a problem, it proceeds to solve it without the need for human participation [7].

AI is the simulation or combination of machine learning and deep learning, the application of which results in deliberate outcomes [5,6]. Furthermore, the use of AI is pervasive and primarily focuses on numerous disciplines in all industries [5,6]. AI is involved in many aspects of the healthcare industry, including patient recordkeeping, medical diagnosis, surgical assistance, and treatment. Additionally, many earlier studies reported that AI is found to have multiple roles in healthcare, for example, AI in improving human decision-making and efficiency; the role of AI in disease conditions, such as radiology, neurosurgical imaging, skin lesions, tumors, chest pain, neurological diseases such as Alzheimer’s disease, and also in the diagnosis of breast cancer; drug discovery; therapy selection, especially in patients with comorbid conditions and on multiple treatments; an increase in the efficiency of human decision-making; and an increase in the effectiveness of human in providing effective care and treatment [3,5,6,8,9].

Several studies internationally revealed that AI has a positive impact on their profession or their workflow [9,10,11,12,13,14,15,16,17,18,19]. Many countries have already adopted the usage of AI [1,5,6,11,20], with the United States, Australia, Canada, and the Chinese healthcare systems [20,21,22,23,24] being among them. For instance, in Canada, a recent study among healthcare students revealed that students projected that AI technology would have an impact on their jobs within the next decade and expressed optimism about the emerging role of AI in their particular disciplines [23]. AI attitudes differed by discipline [23]. Even students who were hostile toward AI recognized the importance of incorporating a rudimentary understanding of AI into their curricula [23]. On the other hand, the Chinese State Council has issued a guideline on AI development, indicating that the widespread use of AI will raise the level of precision in medical services and attain intelligent medical care [24]. In Saudi Arabia, a recent survey conducted across the country revealed that 7% of Saudi radiology residents used artificial intelligence in their daily work [5], which indicates that the use of AI in various aspects of healthcare will become more widespread in the future. Therefore, knowledge and perceptions about AI and machine learning (ML) help for when AI comes into use, since professionals will be using them soon, and they are expected to play an important role in their clinical workflow. A lack of knowledge among healthcare professionals regarding AI and ML may translate into poorer patient outcomes due to a lack of understanding about selecting tools that add value and integrating these into patient care.

It is commonly known that pharmacists play a variety of important roles in healthcare, including patient counseling and education. Pharmacists are typically the first healthcare professionals that people turn to for medical advice, since they play a crucial part in the safe and effective use of drugs [25]. According to this pharmaceutical perspective, undergraduates are future working pharmacists, and demonstrating enough knowledge by the time they graduated helps them both at their place of employment and in getting great grades. During pharmacy education at the undergraduate level, AI is not frequently covered. The current accreditation criteria do not highlight AI, as healthcare schools are already grappling with a heavy curriculum and are frequently asked to add additional topics and areas of study and they lack professors with the skills to teach this topic. Another possible challenge is that pharmacy schools and educators still do not know the exact emerging role of the pharmacist regarding AI and, hence, are unable to devise teaching approaches [26,27].

Since AI will be used soon and is anticipated to play a significant part in healthcare workflow, having skills in AI and machine learning (ML) would be helpful. Due to a lack of awareness about how to choose tools that provide value and incorporate these into patient care, a lack of knowledge among healthcare workers regarding AI and ML may result in worse patient outcomes. The use of AI has been on the rise globally, as well as locally. In addition, no study has been done to evaluate the awareness, perceptions, and opinions toward artificial intelligence among undergraduate pharmacy students in Saudi Arabia. Therefore, this study focuses on determining awareness, perceptions, and opinions toward artificial intelligence among undergraduate pharmacy students at King Saud University, Riyadh, Kingdom of Saudi Arabia.

## 2. Materials and Methods 

### 2.1. Study Design, Setting, and Population 

**Scheme 1:**
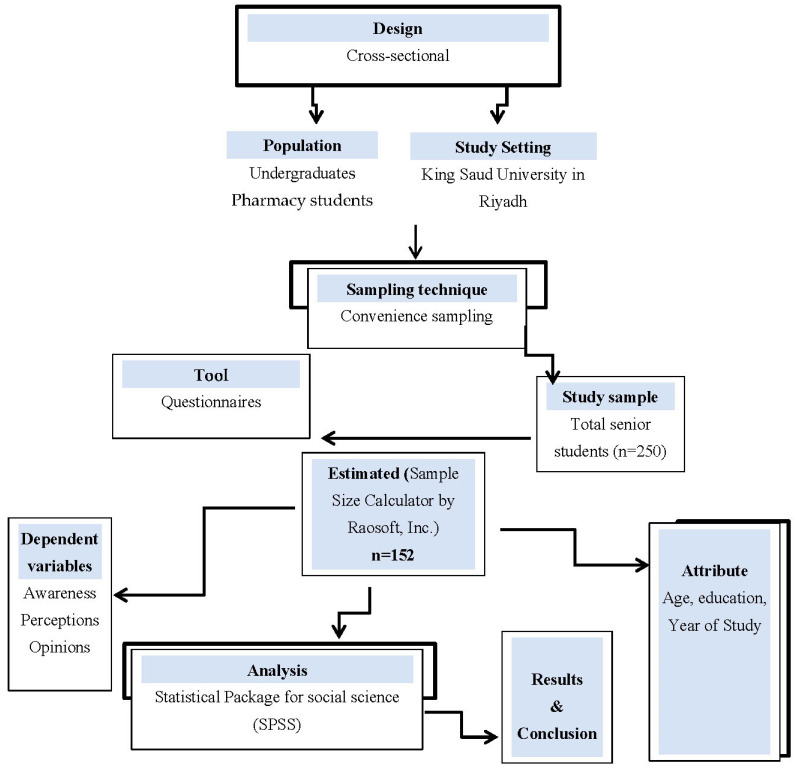
Outline of the research design.

A cross-sectional study was conducted among undergraduate PharmD students at King Saud University College of Pharmacy between December 2022 and January 2023. All students from the first year to the final year studying their pharmacy courses were invited to participate. The detailed design of the study was presented in Scheme 1. Students not studying pharmacy, aged >18 years, and students from other universities were excluded. Before data collection, the study was approved by the ethical committee of the College of Medicine, King Saud University, Riyadh Saudi Arabia. Before beginning the study, students verbally consented after being fully informed. Students who signed the consent form were also included in the study. Additionally, assurances were given to the students that the information would only be utilized for research purposes and that the study would be conducted in strict confidence. Students were also given the option to leave the study at any time.

### 2.2. Designing of the Questionnaires

A questionnaire was developed using the literature [13,14,15,16] to fulfill the study’s aim. It is composed of 3 sections, including students’ characteristics, perceptions of AI, and opinions of AI. Regarding perceptions towards AI, twelve questions were used to assess this that were adopted from other studies [13,14,15,16] after subjecting them to a few modifications. One point per question was rewarded if the question was answered correctly. To assess the opinions on artificial intelligence, a three-point Likert scale assessment tool consisting of 7 variables was adopted from a study [16]. The mean positive perceptions score towards AI was prepared for the perception items. The mean score was prepared by assigning a score of ‘1’ for the correct answers and a score of ‘0’ for the wrong answers. By computing the total items, the mean score was prepared. 

After the initial draft of the questionnaires, it was subjected to content and face validation to determine the suitability, flow, and time taken to answer the questionnaire, with the help of a senior professor and a researcher who were experts in designing and validating cross-sectional studies. In addition, a pilot study was conducted among a randomly selected sample of pharmacy students. The questionnaire reliability test revealed an acceptable level of internal consistency after calculating the Cronbach’s Alpha coefficient based on the answers of only 30 randomly selected undergraduate pharmacy students (12 questions that assess the perceptions of AI (Cronbach’s Alpha = 0.82) and 17 questions that assess the opinions an AI (Cronbach’s Alpha = 0.78)). 

### 2.3. Statistical Analysis 

Following the data collection, the questionnaire was checked for accuracy and completeness. The data were analyzed using SPSS statistical software package, version 26 (SPSS Inc. Armonk, New York, United States). A descriptive analysis was used to estimate frequencies, and the chi-square test was utilized to assess group differences. Results with a *p*-value of <0.05 were considered statistically significant.

## 3. Results

### 3.1. Sociodemographic Characteristics

A total of 157 pharmacy students completed the questionnaires. Of these, 118 (75.2%) were male and 39 (24.8%) were females. Of the respondents, 101 (64.3%) were aged 18–22 years. Of about 42% of the respondents, 65 (41.4%) were in their fourth year of study, while 36 (22.9%) of them were in their fifth year, with 56 (35.7%) of them pursuing an internship. Furthermore, students’ demographic characteristics and professional information are summarized in Table 1.

### 3.2. Awareness of Students about Artificial Intelligence (AI)

**Figure 1:**
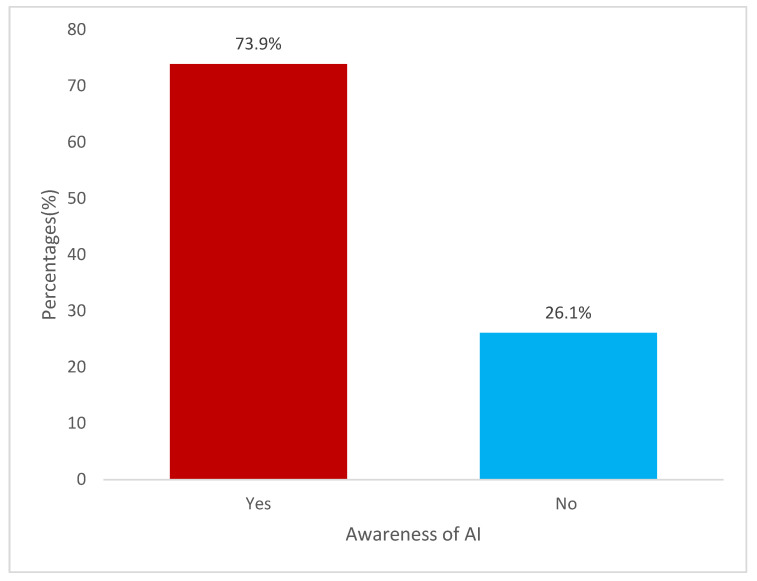
Awareness of AI.

In the second part of this study, we explored the awareness of students about artificial intelligence. Most of the students (*n* = 116; 73.9%) knew about AI (Figure 1). In addition, 69.4% (*n* = 109) of the students thought that AI is a tool that helps healthcare professionals (HCP). However, more than half 57.3% (*n* = 90) of the students were aware that AI would assist healthcare professionals to become better with the widespread use of artificial intelligence in Saudi Arabia. Moreover, 126 (80.3%) did not receive any formal education regarding AI. The detailed frequencies of the students toward an awareness of AI are given in Table 2.

### 3.3. Perceptions of Students about AI 

Table 3 reports the perceptions of students about AI. In this study, slightly less than half (46.9%; *n* = 73) of the students disagreed that AI devalues the medical profession, and 75.1% of the students agreed that AI reduces errors in medical practice, while 77.7% and 63% of the students agreed to the statement that AI facilitates healthcare professionals’ access to information and patients’ access to the service. The majority of the respondents (82.8%) thought that AI enables HCP to make accurate decisions. Almost half of the students (51%, 49%) were neutral, with the statements, that AI increases a patient’s confidence in medicine and allows the patient to increase control over their health. More than half of the students (56.7%) agreed that AI facilitates patient education, while only 28.6% think that AI negatively affects the relationship between healthcare professionals and the patient, although half of the students agreed that artificial intelligence reduces the humanistic aspect of the medical profession. Moreover, 36.3% of the students disagreed with the statement that artificial intelligence violations of professional confidentiality may occur more. The detailed responses of the students on their perceptions of AI are given in Table 3.

### 3.4. Opinions about Artificial Intelligence (AI)

More than half (56.7%, *n* = 89) of respondents agreed that knowledge and skills about artificial intelligence (AI) should be included in the academic curriculum. More than two-thirds of the students think that artificial intelligence (AI) as an application for reducing medication errors should be included, while 70.1% think that training to prevent and solve ethical problems that may arise with artificial intelligence (AI) applications should be included. Moreover, when the study cohort was asked whether they agreed with the statement that there should be a simplified lecture on artificial intelligence, computer use, coding, and Python language, 61.8% of the students agreed that it should be included. When students were asked whether they believed that artificial intelligence (AI) in scientific research should be included, 59.9% (*n* = 94) said that it should be included, while 49% (*n* = 77) said that artificial intelligence (AI) assisted emergency responses. The details of the replies to the opinions of students about artificial Intelligence (AI) are summarized in Table 4.

The mean positive perception score was 29.8 (SD = 9.63; range-0–38). The mean positive perception score was significantly associated with age (*p* = 0.030), year of study *(p* = 0.040), and nationality (*p* = 0.013). The gender of the participants was found to have no significant association with the mean positive perception score (*p* = 0.916). Furthermore, a detailed explanation of the association between the mean positive perception score and the demographics of the participants is given in Table 5. Additionally, the mean positive perception score was not significantly associated with the awareness of AI (*p* = 0.106). 

## 4. Discussion

To the best of our knowledge, this is the first kind of study in Saudi Arabia that aimed to explore the awareness, perception, and opinions of pharmacy students toward artificial intelligence. Not much literature was identified nationally and internationally about the awareness, perception, and opinions of undergraduates toward artificial intelligence in healthcare; however, most of the literature reported was among practicing healthcare professionals and medical students [13,14,15,16,17,18,23,28]. This study would add significant awareness to healthcare students and make the advanced delivery of healthcare to the patients, individuals in the community, and hospital pharmacy and clinical settings in Saudi Arabia and would serve as a reference for the much-needed upcoming studies.

The findings of this study reported that artificial intelligence would not reduce the humanistic aspect of the medical profession, while 23% of them agreed that AI reduces the workforce. These findings were comparable to previous findings published by Jha et al., where the author reported that 24.1% of medical students and doctors disagreed about AI reducing the workforce [29]. Similarly, in this study, 75% (*n* = 118) of the students agreed or strongly agreed that artificial intelligence reduces errors in medical practice, while another recent study from a developing country by Ahmed et al. in 2022 revealed that 3.2% of students and 6.3% of healthcare professionals strongly agreed and 27.1% of students and 30% of doctors agreed that AI implementation reduces the errors in diagnosis and treatment, while 2.4% of students and 4% of healthcare professionals strongly disagreed and 27.9% of students and 33.6% of healthcare professionals disagreed regarding the contribution of AI in reducing medical errors [30].

Although, in this study, 17.8% of the students believed that artificial intelligence replaces the physician, pharmacist, or nurse in the healthcare system, Jha et al.’s the study revealed that over half the respondents agreed that AI would reduce the number of jobs for healthcare professionals [29]. Similarly, Ahmed et al. in 2022 revealed that approximately 70% of medical students and 81.8% of doctors from the study population acknowledged that AI could serve as a practitioner’s aid soon, and most of them did not consider AI as a physician’s replacement but rather a physician’s diagnostic aid [30]. Regarding the inclusion of AI in the pharmacy curriculum, 56.7% of the students agreed to include knowledge and skills about AI, while 61.8% of the students agreed to include a simplified lecture on artificial intelligence, computer use, coding, and Python language. A similar study concluded that 74% of medical students and 83% of physicians agreed to include it in the school curriculum, and 64.9% acknowledged its necessity in radiology, while 59.8% agreed with its use in pathology and the COVID-19 pandemic, respectively [30]. Similarly, another study reported that 91.5% of students in the United States agreed that training in artificial intelligence during medical school would be useful for their future, while 79.4% were excited to use artificial intelligence technologies [31].

In this study, pharmacy students appeared to have good perceptions and opinions about AI and its benefits in healthcare. A study among medical students showed that 50% of the students agreed that they have a good understanding of AI [22], whereas a study on dental students in Saudi Arabia concluded that only 44.2% were aware of the usage of AI in dentistry [28], while a study in Canada among healthcare students concluded that 51.08% were not aware of AI [23]. Encouragingly, the majority of pharmacy students (69.4%) believed AI is a tool that helps HCPs rather than replaces HCPs in the healthcare system. It is a fact that rapid advances in the field of technology will certainly change the practice of HCPs, as routine tasks can be performed faster and more efficiently with the aid of AI. In our study, more than half (57.3%) of the students believed that AI would make the healthcare profession better. According to a study by Teng et al., a positive outlook on AI development in their respective fields confirmed that AI would have a positive impact on their careers [23]. AI works by collaborating with researchers to enhance decision-making processes for existing pharmaceuticals and expanding therapies for various ailments, as well as to speed up clinical trials by identifying suitable patients from several data sources [32]. In pharmacy and hospital settings, AI is being utilized to reduce hospital readmissions and avoid medical errors by evaluating patient data for medical and medication errors, readmission root causes, and other medical data sources [33,34,35]. Furthermore, AI serve more people in a very short period [33,34,35]. Furthermore, the growth of AI technology may result in more rapid and cost-effective healthcare and pharmaceutical research, as well as better service to the general population [33,34,35].

The pharmacist is uniquely positioned to contribute to the advancement of artificial intelligence in healthcare. Furthermore, they should also participate in the validation of AI for clinical use. Additionally, future pharmacists should be ready to adapt to AI use, which may be achieved by additional education programs about AI and its application in the healthcare setting. Furthermore, most students regarded it as essential to include it in the college curriculum and also use studies, seminars, or residency training as the best way to educate students about the use of AI in healthcare.

The mean perception of AI in this study was 29.8 (SD = 9.63; range 0–38). Age, year of study, and nationality all had a significant impact on the mean positive perception score. Furthermore, the mean positive perception score was not significantly related to AI awareness. This could be better explained by the fact that senior undergraduates consistently demonstrate a higher level of awareness or knowledge than juniors and others, as well as the fact that the older an individual is, the higher the level of knowledge and awareness. Furthermore, prior exposure to AI during the graduation process (via a course, congress, seminar, etc.) may have influenced this situation. AI has been an acquainted theme in the medical and healthcare research community for the past few years. However, one of the main challenges has been the lack of familiarity and understanding of this technique among healthcare professionals. This study would add a significant contribution to the implementation of AI and would serve as a reference for the much-needed upcoming studies. The findings could also be used by educational and healthcare institutions to develop appropriate training initiatives to improve AI courses.

The results of this study may have been predisposed to some limitations. Firstly, it was conducted with pharmacy students from only one university. Consequently, it might not be generalized to other student populations in this and other universities in Saudi Arabia. Inconsistent samples across different disciplines are attributable to various factors, as discussed earlier. Due to these differences, we were unable to compare the perceptions and awareness of AI across other disciplines in other health colleges at both the national and international levels and provide country-specific recommendations. Third, because this was an online, self-administered survey, we relied on respondents accurately documenting their responses without the ability to check this, which may have contributed to a potential bias. There are also limitations attributed to the nature of the cross-sectional study, such as the inability to assess causal relationships. Furthermore, recruitment methods were conveniently sought primarily from the institutions to which the researchers were affiliated. Despite these limitations, we believe the study provides useful insights into students’ perspectives, awareness, opinions, and the impact of AI on this population in Saudi Arabia and more. Consequently, we believe the findings are useful in providing further guidance to education and health policymakers.

## 5. Conclusions

In conclusion, the current findings revealed that pharmacy students at a Saudi university in Riyadh appeared to have positive perceptions, awareness, and good opinions towards AI and its use in the healthcare setting. Our results suggested that students must be aware of the new technologies in healthcare such as AI and its progress and its implications. Educational programs about the procedure need to be considered and should focus on the acceptance of such new therapeutic management. More efforts may be needed to ensure that face-to-face courses are subsequently delivered in a suitable online format with pertinent assessments and tasks without overload.

## Figures and Tables

**Table 1:** The demographic characteristics of the students (*n* = 157).

Variables	Frequency (*n*)	Percentage (%)
Gender		
Male	118	75.2%
Female	39	24.8%
Age		
18–22	101	64.3%
23–25	52	33.1%
26–30	4	2.5%
Nationality		
Saudi	153	97.5%
Non-Saudi	4	2.5%
Level/year of study		
Fourth-year	65	41.45
Fifth year	36	22.9%
Internship	56	35.7%

**Table 2:** Opinions of students about artificial intelligence (AI) (*n* = 157).

Variables	Frequency	Percentage
Do you think that Artificial intelligence will replace the physician, pharmacist, or nurse in the healthcare system?		
Agree	28	17.8%
Disagree	20	12.7%
It is a tool that helps healthcare professionals	109	69.4%
What is your opinion, if artificial intelligence is widespread in Saudi Arabia?		
Risk of losing jobs with the introduction of robots (Artificial intelligence) with the decrease in the need for employees	39	24.8%
Healthcare professionals will be better with the widespread use of artificial intelligence.	90	57.3%
The choice of specialization Field will be influenced by how artificial intelligence is used in that Field	16	10.2%
I don’t know	12	7.6%
Have you received any formal education about artificial intelligence?		
Yes	16	10.2%
No	126	80.3%
Received training over the internet	4	2.5%
Through seminars and presentations	11	7.0%

**Table 3:** Perceptions of the students about AI (*n* = 157).

Variables	Strongly Agree *n* (%)	Agree *n* (%)	Neutral *n* (%)	Disagree *n* (%)	Strongly Disagree *n* (%)
Artificial intelligence (AI) devalues the medical profession	24 (15.3%)	12 (7.6%)	48 (30.6%)	52 (33.1%)	21 (13.4%)
Artificial intelligence (AI) reduces errors in medical practice	41 (26.1%)	77 (49.0%)	24 (15.3%)	8 (5.1%)	7 (4.5%)
Artificial intelligence (AI) facilitates patients’ access to the service	39 (24.8%)	60 (38.2%)	46 (29.3%)	8 (5.1%)	4 (2.5%)
Artificial intelligence (AI) facilitates healthcare professionals’ access to information	66 (42.0%)	56 (35.7%)	35 (22.3%)	0 (0%)	0 (0%)
Artificial intelligence (AI) enables healthcare professionals to make more accurate decisions	60 (38.2%)	70 (44.6%)	27 (17.2%)	0 (0%)	0 (0%)
Artificial intelligence increases patients’ confidence in medicine	16 (10.2%)	41 (26.1%)	80 (51%)	15 (9.6)%	5 (3.2%)
Artificial intelligence facilitates patient education	17 (10.8%)	72 (45.9%)	48 (30.6%)	11 (7.0%)	9 (5.7%)
Artificial intelligence negatively affects the relationship between healthcare professionals the patient	22 (14%)	23 (14.6%)	72 (45.9%)	28 (17.8%)	12 (7.6%)
Artificial intelligence damages the trust which is the basis of the patient-healthcare professional’s relationship	25 (15.9%)	27 (17.2%)	63 (40.1%)	30 (19.1%)	12 (7.6%)
Artificial intelligence reduces the humanistic aspect of the medical profession.	33 (21%)	46 (29.3%)	33 (21%)	29 (18.5%)	16 (10.2%)
Artificial intelligence violations of professional confidentiality may occur more	12 (7.6%)	32 (20.4%)	56 (35.7%)	36 (22.9%)	21 (13.4%)
Artificial intelligence allows the patient to increase his control over his health	13 (8.3%)	32 (20.4%)	77 (49%)	27 (17.2%)	8 (5.1%)

**Table 4:** Opinions of students about artificial Intelligence (AI).

Variables	Should Be Included *n* (%)	Not Sure *n* (%)	I Don’t Know *n* (%)
Knowledge and skills in Artificial intelligence (AI)	89 (56.7%)	52 (33.1%)	16 (10.2%)
Artificial intelligence (AI) as an application for reducing medication errors	97 (61.8%)	40 (25.5%)	20 (12.7%)
Training to prevent and solve ethical problems that may arise with Artificial intelligence (AI) applications	110 (70.1%)	24 (15.3%)	23 (14.6%)
A simplified lecture on Artificial intelligence, Computer use, Coding, Python language	97 (61.8%)	36 (22.9%)	24 (15.3%)
Artificial intelligence (AI) applications that will increase patients’ control over their health	104 (66.2%)	45 (28.7%)	8 (5.1%)
Artificial intelligence (AI) in scientific research	94 (59.9%)	48 (30.6%)	15 (9.6%)
Artificial intelligence (AI) assisted emergency responses	77 (49%)	73 (46.5%)	7 (4.5%)

**Table 5:** Association between the mean positive perception score of AI and characteristics of the participants.

Participants Characteristics	Mean	Std. Deviation (Std)	*F-*Value	*t*-Value	*p-*Value
Gender					
Male	8.69	1.42	-	0.011	0.916 *
Female	8.92	1.54			
Age					
18–22	8.71	1.46			
23–25	8.96	1.39			
26–30	7.00	0.00	3.602	-	0.030
Level/year of study					
Third year	8.46	1.45			
Fourth-year	9.22	1.33	3.293		
Internship	8.78	1.46		-	0.040
Nationality					
Saudi	8.77	0.00	-		
Non-Saudi	8.00	1.46		6.265	0.013 *
Are you aware of artificial intelligence (AI)?					
Yes	8.68	1.36			
No	8.95	1.67	-	2.640	0.106 *

* Independent sample *t* test.

## Data Availability

The data will be available from the correspondence author upon the request.
